# Potential and efficiency of statistical learning closely intertwined with individuals’ executive functions: a mathematical modeling study

**DOI:** 10.1038/s41598-020-75157-8

**Published:** 2020-11-02

**Authors:** Jungtak Park, Hee-Dong Yoon, Taehyun Yoo, Minho Shin, Hyeon-Ae Jeon

**Affiliations:** 1 Department of Brain and Cognitive Sciences, Daegu Gyeongbuk Institute of Science and Technology (DGIST), Daegu, Republic of Korea; 2 Convergence Research Advanced Center for Olfaction, Daegu Gyeongbuk Institute of Science and Technology (DGIST), Daegu, Republic of Korea; 3 Partner Group of the Max Planck Institute for Human Cognitive and Brain Sciences at the Department of Brain and Cognitive Sciences, DGIST, Daegu, Republic of Korea

**Keywords:** Human behaviour, Cognitive neuroscience, Learning and memory

## Abstract

Statistical learning (SL) is essential in enabling humans to extract probabilistic regularities from the world. The ability to accomplish ultimate learning performance with training (i.e., the potential of learning) has been known to be dissociated with performance improvement per amount of learning time (i.e., the efficiency of learning). Here, we quantified the potential and efficiency of SL separately through mathematical modeling and scrutinized how they were affected by various executive functions. Our results showed that a high potential of SL was associated with poor inhibition and good visuo-spatial working memory, whereas high efficiency of SL was closely related to good inhibition and good set-shifting. We unveiled the distinct characteristics of SL in relation to potential and efficiency and their interaction with executive functions.

## Introduction

Statistical learning (SL) is an implicit mechanism that requires learners to extract probabilistic regularities from the environment^1–7^. It is a critical process in our daily lives based on the fact that, through SL, learners grasp probabilistic regularities to predict upcoming events and prepare appropriate actions effectively. It has been examined across tasks using various types of stimuli, including visual stimuli^8^, tactile stimuli^9^, non-linguistic sounds^10^, auditory syllables^11^, and action segmentation^12^. SL has been scrutinized by an alternating serial reaction time (ASRT) task in which odd-numbered visual stimuli generate a fixed motor sequence and even-numbered visual stimuli establish random sequences such that participants learn the motor sequence implicitly^13–16^.

The effect of SL, being defined as the difference in learning performance between probable targets (i.e., frequently occurring stimuli) and improbable targets (i.e., infrequently occurring stimuli)^15,17,18^, is known to be related to the frontal lobe of the brain that has been intertwined with higher-order cognitive functions, particularly executive functions^14,15,19–21^. For example, the effect of SL was negatively correlated with a composite score that is an average value of normalized scores of several neuropsychological tests to assess executive functions^19^. An electroencephalography (EEG) study found a dynamic human brain network, showing that a functional connectivity of frontal areas was negatively correlated with individuals’ SL performance^15^. Even though these studies posit a close relationship between SL and the executive functions, there is still a limited understanding of SL. Executive functions include various cognitive processes such as response inhibition, set-shifting, and working memory^22–25^. From these processes, which one is the most pertinent to SL remains unclear. Considering that executive functions are integral to high-level cognitive processes in humans, it is worth scrutinizing each individual process of executive functions in relation to SL.

Learning is not a single entity but is underpinned by several mechanisms^26^. When discussing learning mechanisms, we have to consider two distinct features, that is, the potential of learning and the efficiency of learning. The potential of learning is the ability to accomplish an ultimate learning performance given that learners are provided with all the optimal conditions during the progression of learning^26,27^, and the efficiency of learning is the performance improvement per amount of learning time^26,28^. Interestingly, these two features have been known to be dissociated from each other^26^. For example, even though older adults required more time for motor skill acquisition compared to younger adults, their final performance was comparable to that of younger adults^29,30^. This indicates that, even though the older and younger adults needed different learning times (implying different learning efficiency) to achieve a comparable level of performance, their final levels of performance were similar (implying similar learning potential). Potential and efficiency are of great importance in learning. However, no study has conducted an investigation with respect to these two features, particularly in SL. Therefore, in the present study we distinguished the efficiency of learning from the potential of learning in SL and examined their relations to executive functions, hoping to unveil the contribution of several processes of executive functions (i.e., response inhibition, set-shifting, and working memory) to both the potential and efficiency of SL.

We suggest mathematical modeling as a proper way to estimate individuals’ potential and efficiency of learning objectively and quantitatively. Mathematical modeling formally describes a part (or parts) of cognition in a simplified fashion by converting problems or ideas that should be identified in the experiment into mathematical representations, using mathematical formulations^31,32^. Mathematical modeling, by providing precise quantification, describes assumptions about how observed data is generated and developed^33^ such that it helps better understanding and clarification of research questions or theories than qualitative descriptions^32,34^. In this approach, a model comparison is crucial to select the best model to describe empirical data and to predict its possible changes more precisely^35^. Researchers have used mathematical modeling in describing the benefits from practice in learning and found the exponential function^36^ to be the standard equation to describe and predict the improvement in learning performances^36–40^. In the present study, we focused on SL and aimed to find the best model to reflect SL performances by testing goodness-of-fit of the exponential function in comparison with power or linear functions (as control models)^41–44^. According to a formula of the exponential function, a learning rate decreases and eventually stays consistent even with increasing practice^36^. Using this function, we estimated the potential and efficiency of SL and investigated the dynamic changes in SL performance (for details, see “Methods”). Using mathematical modeling, we objectified abstract psychological phenomena (i.e., the potential and efficiency of SL) as being measurable estimates such that we were able to provide a prevailing account of how these two features reconcile with various executive functions during SL.

**Figure 1:**
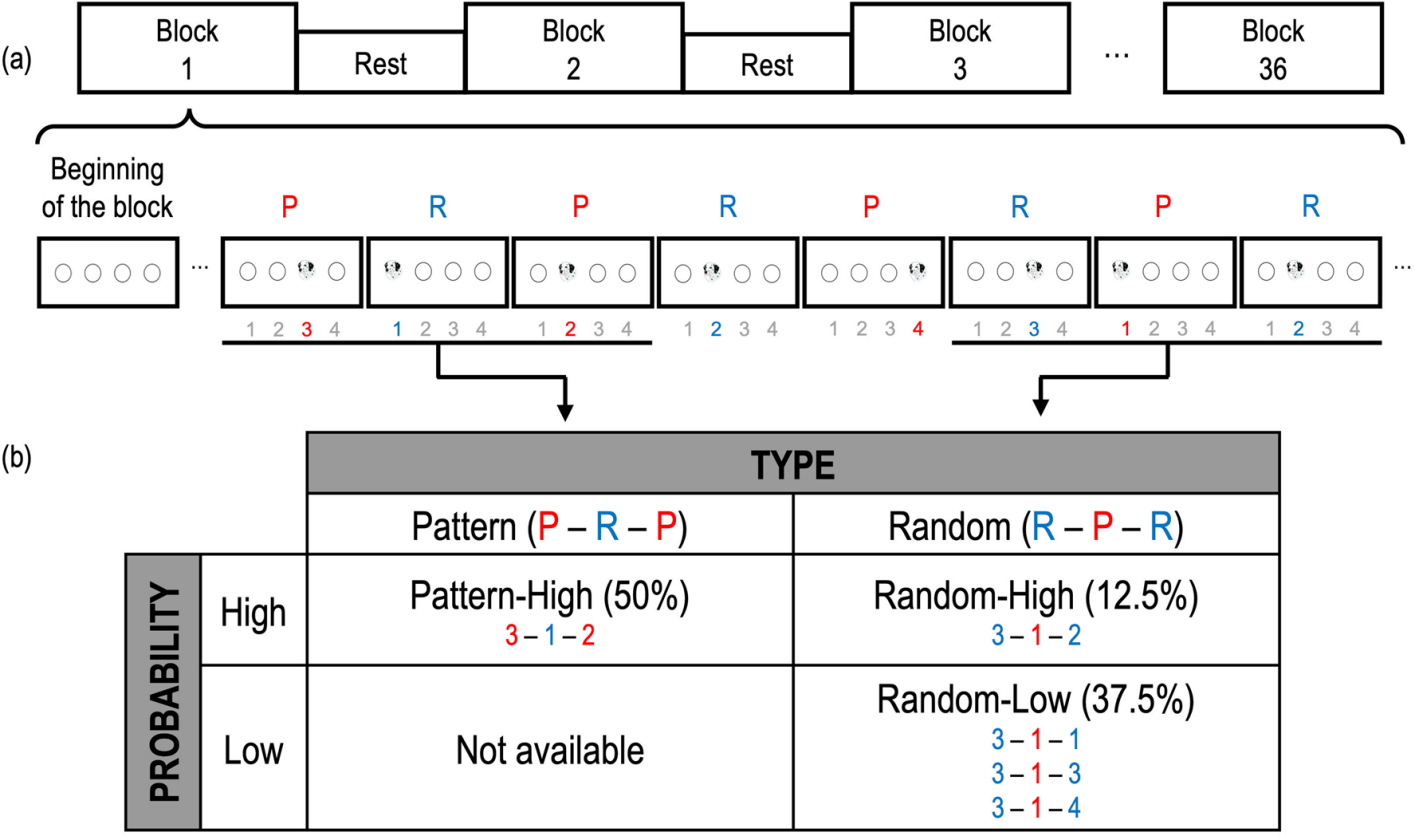
Design of ASRT task. (**A**) The experiment consisted of 36 blocks with rest blocks in between. A block started with four empty circles shown on the screen for 200 ms, then a trial started with a dog’s face being shown in one of the four circles for 500 ms. Participants were asked to push one of four buttons corresponding to the target (a dog’s face) position. A block consisted of 85 trials with an inter-trial-interval (ITI) of 120 ms. Pattern trials (P) and random trials (R) were shown in an alternating order, which established an alternating serial sequence composed of eight target trials. For example, the figure showed the sequence of 31224312 where red numbers (3–2–4–1–) were repeated 10 times within a block and were alternated with blue random numbers. The red and blue colors are displayed here for an easy explanation and were not shown in the actual experiment. (**B**) We combined three trials into a triplet so that alternating serial sequences generated three conditions such as Pattern-High, Random-High, and Random-Low. Probability is based on the number of occurrences of a triplet, that is, high probability or low probability. Type is based on a triplet composed of either P–R–P [pattern type] or R–P–R [random type]. In the example, ‘3–1–2’ is Pattern-High, which indicates that 2 (pattern trial) is highly predictable after 3 (pattern trial) and 1 (random trial). Random type can be either high probability (Random-High) or low probability (Random-Low). Some triplets in the random type (3–1–2, 12.5%) could also be observed in the pattern type (3–1–2, 50%), and thus they were referred to Random-High. The rest of the triplets in random type are Random-Low, because they had a low probability of occurrence [37.5% (12.5% $\times$ 3)].

Out of several executive functions, we aimed to elucidate which of them had the most influence on the potential and efficiency of SL. To this end, we first examined the effect of SL in an ASRT task through the difference in learning performance between probable targets and improbable targets. The ASRT task had three different conditions: pattern type with a high probability condition (Pattern-High), random type with a high probability condition (Random-High), and random type with a low probability condition (Random-Low) (Fig. 1). Because the Random-High and Random-Low conditions were separated only by probability (i.e., a high probability and a low probability) with the same type (i.e., random type), the comparison between these two conditions—SL scores—enabled us to evaluate the effect of SL. Second, in virtue of mathematical modeling, we quantified participants’ potential and efficiency of SL by model fitting with SL scores. Third, we calculated correlation coefficients between the scores of neuropsychological tests for executive functions and the potential and efficiency of SL, presenting a novel and precise explanation of how these two overarching features of SL are mediated by various executive functions. To foreshadow the core findings, exponential function was selected as the best model to represent SL scores. Accordingly, by measuring the potential and efficiency of SL using the exponential model, we found that inhibitory control was negatively correlated with the potential of SL and positively correlated with the efficiency of SL. Furthermore, good set-shifting was associated with a high efficiency of SL and good visuo-spatial working memory was related to a high potential of SL. Our study makes significant progress towards unraveling the overarching roles of both the potential and efficiency of SL, which are closely interwoven with various executive functions.

## Results

### Descriptive statistics of data from the ASRT task

Participants showed high accuracy (total: 91.9%, SD = 2.0; Pattern-High: 92.7%, SD = 1.0; Random-High: 92.9%, SD = 1.6; Random-Low: 90.2%, SD = 1.8) and fast reaction times (RTs) (Total: 285.0 ms, SD = 6.1; Pattern-High: 284.1 ms, SD = 4.1; Random-High: 280.5 ms, SD = 4.3; Random-Low: 290.3 ms, SD = 5.1). These results indicate that participants successfully performed the ASRT task.

### Effect of SL and effect of type through multiple linear regressions

We hypothesized that an increase in performance differences between Random-High and Random-Low occurs through the progression of SL, and thus an interaction between conditions (i.e., Random-High and Random-Low) and learning time (i.e., block order) predicts the effects of SL. We used a multiple linear regression^45^ with two independent variables of conditions (i.e., Random-High and Random-Low) and learning time (i.e., block order), and included the interaction between the conditions and learning time for the prediction of SL effects in behavioral performances (i.e., accuracy and RTs). The results are shown in the Table 1. The effect of SL (i.e., interaction term) significantly affected RT [${t}_{(68)}$ = 2.7, *P* = 0.008, 95% confidence interval (CI_95%_) = 0.8 to 5.0], while a marginal influence was observed for accuracy [${t}_{(68)}$ = − 2.0, *P* = 0.053, CI_95%_ = − 0.010 to 7.2 $\times {10}^{-5}$]. These results showed that the participants’ success in capturing probabilistic sequences in the ASRT task was well represented by the RT data. Moreover, since participants’ accuracy remained very high across all the conditions, we used only RT data for further analyses. We also examined the effect of type (Table 2) that induces the performance difference between Pattern-High and Random-High over the learning time. However, no significant effect of type was found in our data [accuracy: ${t}_{(68)}$ = − 1.3, *P* = 0.190, CI_95%_ = − 0.008 to 0.002; mean RT: ${t}_{(68)}$ = − 0.6, *P* = 0.530, CI_95%_ = − 2.6 to 1.4].

**Table 1:** Effect of SL measured by a multiple linear regression model (Random-High vs. Random-Low).

		Coefficients				
		$\beta$	*SE*	*t*	*P*	CI_95%_
Accuracy	(Constant)	0.929	0.002	520.0	*P* = 3.9 $\times$ 10^–124^	0.925 to 0.932
	Condition	− 0.026	0.003	− 10.5	*P* = 8.4 $\times$ 10^–16^	− 0.031 to − 0.021
	Block order	− 0.011	0.002	− 6.2	*P* = 4.2 $\times$ 10^–8^	− 0.015 to − 0.007
	Interaction	− 0.005	0.003	− 2.0	*P* = 0.053	− 0.010 to 7.2 $\times$ 10^–5^
RT (ms)	(Constant)	280.5	0.751	373.7	*P* = 2.2 $\times$ 10^–114^	279.0 to 282.0
	Condition	9.8	1.062	9.3	*P* = 1.2 $\times$ 10^–13^	7.7 to 11.9
	Block order	− 1.0	0.751	− 1.3	*P* = 0.197	− 2.5 to 0.5
	Interaction	2.9	1.062	2.7	*P* = 0.008	0.8 to 5.0

	Model summary					
	${F}_{(\mathrm{3,68})}$	*P*	Adj.${R}^{2}$	${AIC}_{c}$	$BIC$	
Accuracy	75.95	1.1 $\times$ 10^–21^	0.760	− 444.4	− 435.9	
RT	31.27	8.1 $\times$ 10^–13^	0.561	425.5	434.0	

*SE* standard error, *CI* confidence interval, *Adj.*${R}^{2}$ adjusted ${R}^{2}$, ${AIC}_{c}$ corrected Akaike information criterion, $BIC$ Bayesian information criterion.

**Table 2:** Effect of type measured by a multiple linear regression model (Pattern-High vs. Random-High).

		Coefficients				
		$\beta$	*SE*	*t*	*P*	CI_95%_
Accuracy	(Constant)	0.927	0.002	571.6	*P* = 6.3 $\times$ 10^–127^	0.924 to 0.930
	Condition	− 0.002	0.002	0.7	*P* = 0.468	− 0.003 to 0.006
	Block order	− 0.008	0.002	− 4.9	*P* = 5.5 $\times$ 10^–6^	− 0.011 to − 0.005
	Interaction	− 0.003	0.002	− 1.3	*P* = 0.190	− 0.008 to 0.002
RT (ms)	(Constant)	284.1	0.703	404.1	*P* = 1.1 $\times$ 10^–116^	282.7 to 285.5
	Condition	− 3.6	0.994	− 3.6	*P* = 5.2 $\times$ 10^–4^	− 5.6 to − 1.6
	Block order	− 0.4	0.703	− 0.5	*P* = 0.620	− 1.8 to 1.1
	Interaction	− 0.6	0.994	− 0.6	*P* = 0.530	− 2.6 to 1.4

	Model summary					
	${F}_{(\mathrm{3,68})}$	*P*	Adj.${R}^{2}$	${AIC}_{c}$	$BIC$	
Accuracy	23.71	1.3 $\times$ 10^–10^	0.490	− 458.3	− 449.8	
RT	5.15	2.9 $\times$ 10^–3^	0.149	416.1	424.6	

*SE* standard error, *CI* confidence interval, *Adj.*${R}^{2}$ adjusted ${R}^{2}$, ${AIC}_{c}$ corrected Akaike information criterion, $BIC$ Bayesian information criterion.

### Mathematical modeling of SL scores

We examined the effect of SL using SL scores that are defined as absolute values of the performance differences between Random-High and Random-Low (see “Data analysis” for details). To find the best model to delineate participants’ SL scores, we investigated three different models known to represent learning progress^41–44,46^. Using maximum likelihood estimation (MLE)^47,48^, we fitted an exponential model $[y = {w}_{1}(1-{e}^{-\frac{x-{w}_{2}}{{w}_{3}}})]$, a power model $[y = {w}_{1}{(x-{w}_{2})}^{{w}_{3}}$], and a linear model [$y = {w}_{1}(x-{w}_{2})+{w}_{3}$] to all the participants’ SL scores. The parameters $w$ are different estimated parameters in the three learning models. Values of the parameters, corrected Akaike information criterion ($AICc$)^49^, and Bayesian information criterion ($BIC$)^50^ for the learning models are shown in Table 3. To select the best model, we calculated the $\Delta AICc$ and the $Bayes \, factor$ (see “Data analysis” for details) for all models. Based on the scales for interpreting the $\Delta AICc$ and the $Bayes \, factor$ (Table 4)^49,51,52^, the exponential function turned out to be a better model fit than the linear function ($\Delta AICc$ = 7, $Bayes \, factor$ = 54.6) (Table 5). Moreover, since the exponential function had the smallest value of $AICc$ and $BIC$ (Table 3), we concluded that modeling our data with the exponential function worked best, and thus we considered only the exponential model for further analyses.

**Table 3:** Value of parameters, ${AIC}_{c}$, and $BIC$ in mathematical models.

	Models fitted to SL scores		
	Exponential	Power	Linear
Parameter ${w}_{1}$	13.25	3.20	0.27
Parameter ${w}_{2}$	− 0.39	− 0.34	− 0.50
Parameter ${w}_{3}$	10.28	0.41	4.74
*AICc*	12,514	12,517	12,521
*BIC*	12,508	12,511	12,516

*Exponential* an exponential model, *Power* a power model, *Linear* a linear model, ${AIC}_{c}$ corrected Akaike information criterion, $BIC$ Bayesian information criterion.

**Table 4:** Scales for interpreting the $\Delta {AIC}_{c}$ and $Bayes \, factor$ for model ${M}_{1}$ against model ${M}_{0}$.

$\Delta {AIC}_{c}$	$Bayes \, factor$	Interpretation
< 2	< 1	Substantially supports the ${M}_{0}$
2–4	1–3	Not worth more than a bare mention
4–7	3–20	Positively supports the ${M}_{1}$
7–10	20–150	Strongly supports the ${M}_{1}$
> 10	> 150	Very strongly supports the ${M}_{1}$

${AIC}_{c}$ corrected Akaike information criterion.

**Table 5:** Comparison of model fittings.

${M}_{1}$ vs.${M}_{0}$	$\Delta {AIC}_{c}$	$Bayes \, factor$
Exponential vs. linear	7	54.6
Power vs. linear	4	12.2
Exponential vs. power	3	4.5

${AIC}_{c}$ corrected Akaike information criterion.

**Figure 2:**
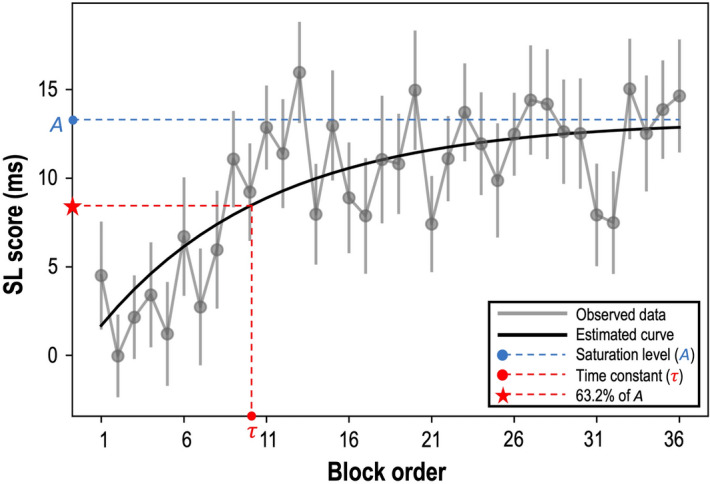
The increase of SL scores in all the participants over time. X-axis and y-axis indicate the block order and SL score, respectively. Gray dots represent averaged SL scores of all the participants and the black solid line is an estimated curve from the exponential model. The blue dashed line exhibits the saturation level of SL scores ($A$). The $\tau$ (the red circle) is a time point to reach approximately 63.2% of $A$ (the red star). Error bars indicate the standard error of the mean.

It is challenging to quantify psychological factors (i.e., the potential and efficiency of learning) using objective measures (i.e. accuracy and RTs). However, through mathematical modeling we were able to investigate the change of SL over time using the estimated learning curve in the exponential model [$y= A\times (1-{e}^{-(x-{x}_{0})/\tau }$)] (Fig. 2). Here, the potential, efficiency, and starting point of SL in all the participants were successfully quantified by the saturation level of the SL score ($A$), the time constant ($\tau$), and the x-intercept (${x}_{0}$), respectively. The estimated equation for SL scores was: $\mathrm{y}= 13.25\times (1-{e}^{-\frac{x+0.39}{10.28}} )$. This indicated that participants’ saturation level of SL scores was 13.25 ms ($A$ = 13.25) and that the SL scores reached the 63.2% $(\approx 1-\frac{1}{e} )$ of the curve amplitude in the 10th block ($\tau$ = 10.28), which means that if the SL scores continue to increase with its initial learning rate, the SL scores would reach its saturation level ($A=13.25 ms$) after the 10^th^ block ($\tau$ = 10.28). The time constant ($\tau$) is a deterministic factor of efficiency in a systerm^53–59^. Arbitrary large $\tau$ or small $\tau$ represent the slow gain or the fast gain respectively to reach the saturation level of the estimated SL scores. The x-intercept was almost zero (${x}_{0}$ = − 0.39), implying that participants had already started to learn the probabilistic associations of the sequences from the beginning of the ASRT task. Since the individual difference in the starting point of SL is not our main concern, no further discussion will be provided on the starting point of SL.

### Correlation analysis

We were interested in how various executive functions influenced each individual’s potential and efficiency of SL. Therefore, we tested participants by using various neuropsychological tests (see Supplementary method). We subsequently correlated the test scores with the individually estimated saturation level of SL scores ($$A$$) as the indication of the potential of SL, and with the time constant ($$\tau $$) as the indication of the efficiency of SL, using Kendall’s tau (Table 6). Across all participants, the potential of SL ($$A)$$ showed significant positive correlation with the scores of the Corsi block-tapping test [Forwards; CBT(F)] (r = 0.268, *P* = 0.028), indicating that participants with better visuo–spatial working memory had a higher potential of SL. The potential of SL ($$A)$$ also showed a significant positive correlation with the scores of the attention network test (ANT) (r = 0.259, *P* = 0.019). Since the higher scores in ANT indicates worse performance in inhibition, this result notes that participants with poor inhibition demonstrated a higher potential of SL.

**Table 6:** Correlations between the scores of neuropsychological tests and the saturation level of SL score ($A$) and the time constant ($\tau$).

		Scores of participants’ neuropsychological tests									
		Category	Letter	CST (F)	CST (B)	CBT (F)	CBT (B)	WCST	Stroop	ANT	GNG
Saturation level of SL score ($A$)	*r*	− 0.038	0.003	0.003	0.076	0.268	0.009	0.089	− 0.087	0.259	− 0.040
	*P*	0.735	0.981	0.981	0.510	0.028	0.942	0.459	0.428	0.019	0.718
Time constant *(*$\tau$*)*	*r*	− 0.031	0.024	− 0.066	0.029	0.172	− 0.133	0.244	− 0.013	0.242	− 0.019
	*P*	0.787	0.833	0.570	0.804	0.167	0.281	0.047	0.907	0.031	0.870

*Category* Category fluency test, *Letter* Letter fluency test, *CST* Counting span test, *CBT* Corsi block-tapping test, *WCST* Wisconsin card sorting test, *Stroop* Stroop test, *ANT* Attention network test, *GNG* Go/No-go test, *(F)* forwards, *(B)* backwards.

The efficiency of SL ($\tau$) was positively correlated with the scores of the Wisconsin card sorting test (WCST) (*r* = 0.244, *P* = 0.047) and ANT (r = 0.242, *P* = 0.031). Lower scores in WCST and ANT imply better set-shifting and better inhibition, respectively. Similarly, a lower value of the efficiency of SL ($\tau$) also indicates better efficiency of SL. Therefore, the results indicate that people with good set-shifting ability and good inhibitory control performed the ASRT task more efficiently, achieving better SL as learning progressed.

To summarize, individuals with good inhibitory control showed high efficiency, but they seemed to be less competent in their potential of SL. With regard to visuo-spatial working memory and set-shifting, both functions turned out to interact positively with the potential and efficiency of SL, respectively.

Additionally, we observed a positive correlation between the potential of SL ($$A)$$ and the efficiency of SL ($$\tau $$) (*r* = 0.442, *P* = 8.26 $$\times $$ 10^–5^). Since lower values of τ indicate better efficiency of SL, this result indicates that participants who have higher potential tend to show lower efficiency in SL.

## Discussion

We have used mathematical modeling to better understand SL with two critical components of learning, that is, the potential and efficiency of SL and examined how various executive functions (i.e., response inhibition, set-shifting, and working memory) interacted with them. We revealed several important results. First, good inhibition was associated with a low potential of SL, but with a high efficiency of SL. Second, good set-shifting was closely related to a high efficiency of SL. Last, good visuo-spatial working memory was interconnected with a high potential of SL. In the following, we discuss our findings in depth with respect to the nature of SL, more specifically, when SL is still in progress (the efficiency of SL) and ultimately completed (the potential of SL).

### A comprehensive analysis of SL with potential and efficiency through mathematical modeling

Studies on SL have yielded conflicting results regarding its interaction with executive functions^60–62^. For example, there was no significant correlation between learning performance from an implicit SRT task and executive functions measured by forwards and backwards digit span tests and WCST^63^. Opposingly, significant correlations were observed between learning performance of the ASRT task and composite scores of several neuropsychological tests (a listening span test, a counting span test, and a letter fluency test)^19^, showing an important role of executive functions in SL. One possible explanation for this discrepancy may be derived from failing to examine the two critical components in learning, that is, the potential of learning and the efficiency of learning. Only few studies have scrutinized the dissociation between these two components using a motor learning task^26^, and no one has elucidated them in detail, particularly with respect to SL. Therefore, we adopted mathematical modeling to quantify the potential of SL ($A$) and the efficiency of SL ($\tau$) and interrogated how these two overarching components function in SL and how they interact with executive functions.

### Opposing roles of inhibition between the potential of SL and the efficiency of SL

Inhibition, one of the key abilities of executive functions, is known to substantially interact with the learning process^64,65^. For example, kindergarteners (mean age = 5.8 years, SD = 3.9 months) with better inhibitory control showed more improvement when performing a number line estimation task than those with poorer inhibition^65^, which provides a supporting role of inhibitory control in children learning mathematics. In the same vein, participants who achieved a high score in second language acquisition exhibited good inhibition^64^. These studies support a positive contribution of inhibitory control during mathematics and language learning.

However, if we consider a more specific type of learning, namely SL, it is known to be negatively correlated with executive functions^14,15^. One interesting result is that hypnosis boosted SL performance^14^. The advantage of hypnosis was derived from the reduced functional connectivity between frontal and related brain areas, which led to the disturbance in attentional control and executive functions. Another study also showed that poor executive functions were interconnected with better sequence learning^66^. In line with these studies, we demonstrated that poor inhibitory control (e.g., high scores in ANT) was strongly correlated with a high potential of SL ($A$). To interpret this finding, we have to again look closely into the potential of learning with respect to the ultimate completion of learning. The completion of learning cannot go unnoticed without considering automaticity. Learning is considered to be completed when it becomes automatized with minimal involvement of attention or inhibitory control^67–70^. An early stage of learning usually requires a higher degree of attentional and inhibitory controls. Conversely, a late stage of learning requires less attentional and inhibitory controls and more attributes of automaticity^67,69,71–73^. Previous studies showed that grasping regularities of external stimuli was facilitated when one became automatized in SL with less involvement of executive functions and more responsiveness to statistical probabilities, by weakening attentional control or inhibition^74,75^. Likewise, the present study also provides a consistent result, demonstrating that a high potential of SL, which is associated with the ultimate completion of learning, is attributed to the high level of automaticity in learning and thus characterized by the low levels of attention and inhibitory control.

However, the efficiency of SL yields an opposite result from the potential of SL, showing that people good at executive functions such as set-shifting and inhibitory control demonstrated better efficiency of SL (WCST and ANT in $\tau$ in Table 6). Unlike the potential of SL, which is concerned with the completion of learning, the efficiency of SL is related to the improvement in learning performance with practice, whereby it emphasizes the ongoing progress in SL. This fundamental difference—the completion of SL (the potential) and the ongoing progress in SL (the efficiency)—seems to be the main factor to give rise to the opposing results between the potential and efficiency of SL in terms of executive functions. As learning progresses, one becomes automatized by restructuring variables involved in self-monitoring, error correction, or resolving signal-to-noise processing problems^72,76–78^. This indicates that error correction provides an inextricable link to successful learning. Previous studies showed that error rates resort to individuals’ inhibitory control^79–81^. For instance, people deficient in inhibitory control had difficulty waiting to press a button, being inclined to make more errors^82^. In the Go/No-go test, participants with poor inhibition had difficulty in suppressing an impulse to respond to a ‘No-go’ signal, whereupon they made substantial errors and as a result had trouble with learning^79^. In the present study, our participants achieved a high success rate of more than 90% in all the conditions, which indicates that they progressed in SL through monitoring their own behaviors, inhibiting incorrect button presses, and correcting erroneous behaviors. Acquiring high accuracy coincides with making fewer errors that are also compatible with good inhibition and good error correction. Consequently, those who are better at executive functions—particularly inhibition (ANT) in our correlation analysis—advanced in SL, showing more improvement with better efficiency of SL. It should be noted that, among the neuropsychological tests for inhibition (i.e., ANT, Stroop, and Go/Nogo), only ANT scores were correlated with the efficiency of SL in the present study. The reason for this discrepancy seems to stem from the involvement of spatial attention in ANT, but not in Stroop and Go/No-go. One of the important functions required for the successful performance in ANT is the correct allocation of attention on a target that should be spatially separated from distractors^83,84^. In line with this, our participants also assigned attention to the target position correctly for the successful performance in the ASRT task^85–88^. Resultingly, we argue that the significant correlation between the efficiency of SL and ANT scores may be derived from the involvement of inhibition, specifically in combination with spatial attention.

To summarize, we suggest that the significant interaction between high potential of SL and poor inhibition may be attributed to the fact that the potential deals with the completion of learning that is accompanied by a certain degree of automaticity free from inhibitory control. On the contrary, the efficiency of SL pertains to the progress of SL that requires active involvement of self-monitoring, error correction, and inhibition, and thus may benefit from good executive functions.

### A positive relation between set-shifting and the efficiency of SL

Set-shifting, also known as cognitive flexibility or mental flexibility, designates changing perspectives by virtue of accommodating new requirements or rules and overcoming inertial behavior^82^. Specifically in the motor domain, it is important to be flexible for learners to shift stimulus–response mappings correctly for a given trial during the course of learning^26,89^. Our results support this argument, showing that those who made fewer errors in WCST had good efficiency of SL. The WCST is known to measure the function of set-shifting^90,91^. Learners who make fewer perseverative errors in WCST are competent in set-shifting due to high mental flexibility and less perseveration^25,82,92,93^. Thus, they are proficient at correcting errors, which is beneficial to learning. In the same vein our participants, showing better performance in WCST, were relatively flexible in correcting errors, which enhanced their learning progress in SL and they consequently obtained a high efficiency of SL.

### A positive relation between visuo-spatial working memory and the potential of SL

A series of studies have been conducted to investigate the relationship between working memory and sequence learning^19,46,94^, suggesting that people who have higher working memory spans learn sequences more easily than those with lower ones^13,95^. This argument was investigated in more detail in the present study, focusing on the potential of SL. Coinciding with previous studies, we also found a positive correlation between the Corsi block-tapping test scores (forwards) and potential of SL ($A$). In other words, participants with better visuo-spatial working memory demonstrated a higher potential of SL. This positive relationship may be underpinned in the assertion that good visuo-spatial working memory contributes to SL. As previously mentioned, the positive correlation between ANT and the efficiency of SL explained the critical role of spatial attention in SL, because participants were supposed to allocate their attention correctly to the target position while blocking distractors^85–88^. In the same vein, good visuo-spatial working memory seems to aid in learning alternating serial sequences in our ASRT task, helping participants to learn the target position more accurately. To summarize, based on the correlation results from ANT and the efficiency of SL, and Corsi block-tapping test and the potential of SL, we suggest a supporting role of visuo-spatial function over the course of SL.

### The possible potential–efficiency tradeoff in SL

The positive correlation between the potential of SL (A) and the efficiency of SL (τ) indicates that the more the potential of SL increases, the more the efficiency of SL decreases. This correlation result may be interpreted as a possible potential–efficiency tradeoff in SL. We found a similar idea from the well-known phenomenon, so called, the speed–accuracy tradeoff, which indicates that decisions are made slowly with high accuracy and rapidly with a high error rate^96–99^. Analogously, the ultimate learning performance (i.e., the potential of learning) would be high when the performance improvement per amount of learning time (i.e., the efficiency of learning) happens slowly. However, this argument should be validated more carefully with respect to SL in future studies.

### Limitations

Despite the prevailing account of the exponential function to describe SL in the present study, we should consider fitting other mathematical models to the data. Various types of learning patterns in SL have been noted, such as a gradual learning pattern, a decreasing pattern, or a stepwise pattern^100^, which may involve different cognitive functions or learning strategies^101^. Therefore, a future study should look into possible mathematical models intrinsic to these various SL patterns.

## Conclusion

The current study examined SL from several different viewpoints. First, we emphasized individuals’ potential of learning (i.e., how much one could achieve in learning) and efficiency of learning (i.e., how efficiently one could learn), and applied these two components to SL. Second, we used mathematical modeling such that we could rigorously and objectively quantify individuals’ potential and efficiency in SL and unveiled an appropriate mathematical model to best explain SL, that is, an exponential function. Third, we associated executive functions (e.g., inhibition, set-shifting, and working memory) with SL so that we could elucidate how these abilities interact with individuals’ potential and efficiency. Using two critical components of learning mechanisms, namely potential and efficiency^15,17,18,102–104^, the present study sheds new light on the profound understanding of SL processes.

## Methods

### Participants

Forty-four Koreans (mean age = 20.32 years, SD = 1.35 years; 22 females) participated in the experiment. All were right-handed with normal or corrected-to-normal vision and had no history of neurological disease. Every participant signed an informed consent form prior to the experiment. Four participants’ data were excluded from the analysis due to mild depression and a color vision deficiency. The power analysis and minimum sample size were computed based on previous studies^14,15,19^ and with the use of PASS software (https://www.ncss.com/software/pass/)^105^. A priori power analysis indicated a minimum of 36 participants to reach a power of 0.85 for the Kendall’s tau correlation analysis that would detect a correlation coefficient of 0.5 at the significance level $\alpha$ = 0.05. Therefore, the data of 40 participants were considered to be sufficient for the analysis. This study was approved by the Daegu Gyeongbuk Institute of Science and Technology (DGIST) ethics committee in accordance with the Declaration of Helsinki.

### Procedure

Participants were tested in two sessions over two separate days. In Session 1, they took seven neuropsychological tests [the word fluency tests (category and letter)^106–108^, counting span tests (forwards and backwards)^109^, Corsi block-tapping tests (forwards and backwards)^110^, Wisconsin card sorting test^25,82,92,93^, Stroop test^82,111,112^, attention network test^82^, and Go/No-go test^79^], which are known to assess several executive functions (see Supplementary method). In Session 2, participants performed an alternating serial reaction time (ASRT) task^13–16^. Several tools such as E-Prime 3.0^113^, MATLAB^114^, and Psytoolkit^115,116^ (a web-based environment) were used for running tests. In our ASRT task, no feedback was given to participants regarding their task performance. The ASRT task was composed of 36 blocks, which were alternated with rests (Fig. 1a). Each block started with four empty circles shown in the middle of a gray screen for 200 ms. A trial consisted of a target stimulus (a dog’s face) being presented for 500 ms in one of the four empty circles. Participants were asked to press a button corresponding to the target position as accurately and quickly as possible, using a Chronos button box (Psychology Software Tools Inc, Sharpsburg, PA) with the index and middle fingers of both hands. Between trials, four empty circles were presented for 120 ms as an inter-trial-interval. Each block had 85 trials. A fixated cross was shown for six to eight seconds during rests. Each block took 52.9 s and the entire ASRT task took approximately 38–40 min.

Unbeknown to the participants, we alternated between two kinds of main trials: pattern trials and random trials (Fig. 1a). In the pattern trial, the target (a dog’s face) was presented in a fixed position, whereas in the random trial the target was displayed randomly in one of the four positions. For example, a sequence consisting of eight trials, such as 3r2r4r1r (number: a fixed position in the pattern trial, r: a random position in the random trial), indicates an alternating serial sequence of pattern trials (3_2_4_1_) and random trials (_r_r_r_r). In each block, the alternating serial sequence was repeated 10 times, and thus in total 360 times (10 times $\times$ 36 blocks) in the experiment. A specific pattern in the sequence was determined by an order of permutation (e.g., 1r2r3r4r, 1r2r4r3r, …, 4r3r2r1r) for each participant so that the number of occurrences of every alternating serial sequence was counterbalanced across participants. After the ASRT task, participants were asked if they noticed a regular pattern during the experiment. Nobody reported regularities, which indicated that participants did not recognize the structure of the alternating serial sequence explicitly. The first five trials in each block were considered to be warm-up trials with targets in random positions and were not included in analyses.

Three different conditions were constructed by combining type (Pattern vs. Random) and probability (High vs. Low): Pattern-High, Random-High, and Random-Low (Fig. 1b). As for the type, a triplet was composed of three trials that were classified as either a pattern type triplet or a random type triplet^117^. For example, 3r2, 2r4, 4r1, or 1r3 were pattern type triplets because they had two pattern trials (the first and third trials) which were shown regularly in the triplet and only one random trial (the second trial) between the pattern trials. However, r3r, r2r, r4r, or r1r triplets were random type triplets, because they included two random trials (the first and third trials) and only one pattern trial (the second trial) in the middle. With respect to the probability, some triplets (e.g., 312 in Pattern-High and 312 in Random-High in Fig. 1b) were shown more often than others because they were found in both pattern type and random type. On the other hand, some triplets (e.g., 311, 313, and 314) were presented only in the random type. Based on this difference in the occurrences of the triplets, we made a distinction between high probability triplets and low probability triplets. Taken together, we manipulated three conditions by integrating the type with the probability: Pattern-High (pattern type $\times$ high probability), Random-High (random type $\times$ high probability), and Random-Low (random type $\times$ low probability). It is particularly important to note that Random-High and Random-Low were separated solely by the probability of the triplet occurrence, that is, a different probability of occurrences with the same type of triplet. Comparing these two conditions (i.e., Random-High vs. Random-Low) made it possible to investigate the genuine effect of SL, only depending on the probability difference. Pattern-Low is not available in the ASRT task.

Exact probability of occurrence of the triplets was calculated as follows. The pattern type and random type were shown in the same proportion of 1:1. In the random type, Random-High and Random-Low were shown in the proportion of 1:3. Thus, the probabilities of occurrence of the Pattern-High, Random-High, and Random-Low were 50%, 12.5%, and 37.5%, respectively (Fig. 1b). In consequence, high probability triplets and low probability triplets were shown in the proportion of 5:3 [62.5% (50% + 12.5%): 37.5%]. We should also consider the total number of triplets in each condition. Since the number of low probability triplets were three times more than the number of high probability triplets (48 in low probability triplets and 16 in high probability triplets), the high probability triplets were shown five times more than the low probability triplets probabilistically. This probability is calculated as follows Eq. (1):

1 $$ \frac{{5{ }\left( {{\text{probability }}\;{\text{of }}\;{\text{occurence }}\;{\text{of }}\;{\text{high}}\;{\text{ probability }}\;\;{\text{triplets}}} \right)}}{{16 \left( {{\text{number }}\;{\text{of }}\;{\text{high }}\;{\text{probability }}\;{\text{triplets}}} \right)}}:\frac{{3{ }\left( {{\text{probability }}\;{\text{of }}\;{\text{occurence }}\;{\text{of}}\;{\text{ low}}\;{\text{ probability }}\;{\text{triplets}}} \right)}}{{48 \left( {{\text{number }}\;{\text{of }}\;{\text{low }}\;{\text{probability }}\;{\text{triplets}}} \right)}} = 5:1 $$

Notably, a triplet is made of three sequentially presented trials, and a probability of the triplet is determined by the third trial. For example, Fig. 1b shows that even though high and low probability triplets have the identical stimulus positions in the first and second trials (i.e., 3–1– ), they are differentiated by the third trial (high: 3–1–**2**; low: 3–1–**1**, 3–1–**3**, and 3–1–**4**). Therefore, the third trial is the critical element in categorizing the triplets as either high probability or low probability, and thus accuracy and RTs only from the third trials were considered for the analysis.

### Data analysis

#### Investigation of participants’ performances in the ASRT task

We performed all the following analyses using Python 3^118^. Our main interest was to identify the dynamic changes of SL performances over the course of learning time. To do this, first we confirmed if participants successfully achieved SL during the ASRT task with a simple model. The first factor was an effect of probabilistic structure in RTs. We examined this effect by comparing the performances of the high probability and low probability in the same type (i.e., Random-High vs. Random-Low). Since the Random-High and Random-Low have the same type (Random) with different probability (High vs. Low), this factor made it possible to solely examine the pure effects of SL. The second factor was the effect of learning time, which was represented by the block order showing how much learning time had passed. We investigated these two factors (PROBABILITY and BLOCK) independently through the multiple linear regression model^45^ in Eq. (2).

2 $$\mathrm{Performances }\left(\mathrm{accuracy\; or\; RTs}\right) \sim \mathrm{ BLOCK}+\mathrm{PROBABILITY}+\mathrm{ INTERACTION}$$

BLOCK = 1, 2, …, 36 (block order), PROBABILITY = High, Low, INTERACTION = BLOCK $\times$ PROBABILITY.

Here, we supposed that the coefficient of INTERACTION in the model represents the effect of SL over the course of learning time, since the slope of BLOCK would differ between the conditions (Random-High vs. Random-Low) as participants progress in learning.

Likewise, the effect of type (Pattern vs. Random) was investigated in the same probability (High) by comparing the performances of Pattern-High and Random-High. The block order was used to measure the effect of learning time. These two factors were considered in the following multiple linear regression model to see the effect of learning type on RTs Eq. (3).

3 $$ {\text{Performances }}\left( {\text{accuracy or RTs}} \right){ }\sim {\text{ BLOCK}} + {\text{TYPE}} + {\text{ INTERACTION }} $$

BLOCK = 1, 2, …, 36 (block order), TYPE = Pattern, Random, INTERACTION = BLOCK × TYPE.

For all the analyses we used only RTs of correct responses, and the block order was centered for a better fit.

#### Modeling SL scores

To scrutinize the effect of SL together with time, we investigated the learning curve in SL. To this end, we defined SL scores as the following: absolute values of difference in RTs between Random-High and Random-Low. This indicated whether participants learned the statistical probabilities of the triplets^1–3,15^ or not. We calculated the SL scores in every block to investigate the dynamic changes of SL over the course of learning time (block order). Specifically, since an individual’s overall speed of RT could affect the individual SL scores, we adjusted the SL score in each block divided by the mean RT of its corresponding block. We subsequently tested a first-order exponential model $[y = {w}_{1}(1-{e}^{-\frac{x-{w}_{2}}{{w}_{3}}})]$, a power model $[y = {w}_{1}{(x+{w}_{2})}^{{w}_{3}}]$, and a linear model [$y = {w}_{1}(x-{w}_{2})+{w}_{3}]$. Here, y and x indicate the SL scores and order of blocks, respectively. The estimated parameters $w$ are different in each learning model. Maximum likelihood estimation (MLE) was used to fit the SL scores into learning curves^47,48^. To select a model to best explain our SL scores, we used two criteria of goodness-of-fit: the corrected Akaike information criterion ($AICc$)^49^ and the Bayesian information criterion ($BIC$)^50^. Because we did not have many numbers of data point (36 blocks) and participants (40 participants), we used a corrected term ($AICc$) instead of the original $AIC$^49,119^. The equation of $AICc$ and $BIC$ are described below in Eq. (4) and Eq. (5). Here, $k$ is the number of estimated parameters; $n$ is the sample size; and $L$ is the saturated value of the likelihood function for the model.

4 $$AICc=2k\times \frac{n}{n-k-1}-2\times \mathit{log}L $$

5 $$BIC=k\times \mathit{log}n-2\times \mathit{log}L $$

We compared the three models following the scales of Table 4^49,51,52^. Specifically, when we compared the $BIC$ values, we used $Bayes \, factor$^120^. The $Bayes \, factor$ for model ${M}_{1}$ against model ${M}_{0}$ was calculated using the following Eq. (6).

6 $$ Bayes \, factor = e^{{ - 0.5\left( {BIC_{{M_{1} }} - BIC_{{M_{0} }} } \right)}} { } $$

The exponential function turned out to be the best fit for the SL scores compared to other models. This function is described as follows: $y = A\times (1-{e}^{-\frac{x-{x}_{0}}{\tau }})$ ($y$: estimated SL scores, $x$: block order, $A$: saturation level of estimated SL scores, ${x}_{0}$: x-intercept,$\tau$: exponential time constant). This equation is similar to a step response function of a first-order system^53^. In the step response, the saturation level of estimated values—$A$—reflects the predicted ultimate gain^41^. In our model, $A$ represents the potential of SL that indicates participants’ ultimate performance in SL. The x-intercept—${x}_{0}$—reflects the starting point of SL. If the first-order system responds to a step input, the time constant ($\tau$) is defined as a time point to reach $1-\frac{1}{e} (\approx 63.2\%)$ of $A$^53^. In principle, arbitrary large and small $\tau$ represent the slow and fast gain to reach the saturation level of the estimated values, and thus $\tau$ is a reliable factor to determine the efficiency of the system^53–58^. Here, we used the $\tau$ to determine the efficiency of SL that indicates how efficiently participants made progress in SL. When we estimated the value of these parameters in each participant, we used empirical boundaries ($A$: [− 500, 500], ${x}_{0}$: [0, 50], $\tau$: [1, 50], and standard deviation: [0, 30]). In this estimation process, we used the L-BFGS-B algorithm^121,122^ for bound constrained minimization and the initial parameter values of one.

#### Correlation analysis

We calculated Kendall's tau coefficient^123^ and two-sided *p* value between each individual’s neuropsychological test scores and the two parameters—$A$ (potential of SL) and $\tau$ (efficiency of SL)—to explore the relationship between SL and various executive functions. In addition, we conducted a correlation analysis between $A$ (the potential of SL) and $\tau$ (the efficiency of SL) to investigate the possible relationship between them. Since the ${x}_{0}$ (starting point of SL) is not our main interest, we did not address the effect of the starting point in SL. We transformed all the scores into standard z-scores to better fit the normal distribution.

## Supplementary information

Supplementary Information.

## Data Availability

Data in an anonymized form (in accordance to the ethics agreement) and scripts used in data analysis are available upon request.
