# Supplementary material for: Potential and efficiency of statistical learning closely intertwined with individuals’ executive functions: a mathematical modeling study
Source: Sci Rep. 2020 Nov 2;10:18843. doi: 10.1038/s41598-020-75157-8 (PMC7606401; doi:10.1038/s41598-020-75157-8)
Supplement: Supplementary file 1 — Supplementary Information. [file 41598_2020_75157_MOESM1_ESM.docx]

**Supplementary Method**

**Potential and efficiency of statistical learning closely intertwined with individuals’ executive functions: a mathematical modeling study**

Jungtak Park^1^, Hee-Dong Yoon^1,2^, Taehyun Yoo^1^, Minho Shin^1^, and Hyeon-Ae Jeon^1,2,3*^

^1^Department of Brain and Cognitive Sciences, Daegu Gyeongbuk Institute of Science and Technology (DGIST), Daegu, Republic of Korea

^2^Convergence Research Advanced Center for Olfaction, Daegu Gyeongbuk Institute of Science and Technology (DGIST), Daegu, Republic of Korea

^3^Partner Group of the Max Planck Institute for Human Cognitive and Brain ­Sciences at the Department of Brain and Cognitive Sciences, DGIST, Daegu, Republic of Korea

**Corresponding author: *jeonha@dgist.ac.kr**

**Word fluency tests (category and letter).** We conducted two kinds of word fluency tests to measure the ability of individuals’ verbal fluency^1-3^. In the category fluency test, participants produced words that belong to the category of animals and supermarket items. In the letter fluency test, they generated words starting with the Korean consonants ‘ㄱ’, ‘ㅇ’, and ‘ㅅ’^4^. In both tests, participants were required to generate as many words as possible in 60 s. Responses such as repetition, proper nouns, superordinate items, and derivatives were not counted as correct responses^5^. The average number of correct responses was used for scoring.

**Counting span tests (forwards and backwards).** Individuals’ abilities of verbal working memory were measured by counting span tests^6^. In each trial, different numbers of blue circles, blue squares, and yellow circles were randomly displayed on a computer monitor. Participants verbally counted only the blue circles (targets) one by one in every trial. When they had counted the targets, the experimenter changed the display on the screen and participants started the next trial, counting newly presented targets. When a recall cue appeared, participants had to report the numbers of blue circles (targets) of previous trials in the presented order (forward span) or reverse order (backward span). The level, that is, the length of presented trials, varied from two to eight in a set and there were three sets in both the forwards and backwards tests. The final levels—the maximum number of trials correctly produced forwards or backwards in each set—were averaged and used as the score of the counting span test.

**Corsi block-tapping tests (forwards and backwards).** Corsi block-tapping tests assess an individual’s visuo–spatial working memory capacity^7^. Nine purple colored squares were presented on a monitor in a random position, and some of them flickered with a yellow color in a consecutive order. Participants were required to memorize both the locations and the order of the flickering squares and instructed to repeat them by clicking their positions in a forwards or backwards fashion using a computer mouse. The test began with two flickering squares and increased to nine squares. The maximum number of correctly retrieved squares was measured as the score of Corsi block-tapping test.

**Wisconsin card sorting test (WCST).** The ability of set-shifting was measured using the WCST^8-11^. Four cards were shown on the top position of the monitor, and another card was presented at bottom of the screen. Participants were asked to classify the card at the bottom according to three criteria (classification rules) such as color, shape, or number of symbols and to match it to one of the four cards in the upper card deck with a mouse click. They were given feedback after every card selection such that they could adjust the classification rule. The rule changed at every 10^th^ card, but participants were not aware of this so that they had to discover the rule changes themselves^12^. When participants stuck to the previous rule and failed to adjust to a new rule, this was considered to be a perseverative error. The number of perseverative errors was counted as the WCST score.

**Stroop test.** We used a computerized Korean version of the Stroop test to look into the ability of inhibitory control, using four names of colors (red, green, blue, and yellow) displayed in different font colors^11,13,14^. Participants indicated the font color by pressing keys (r, g, b, and y for the colors of red, green, blue, and yellow, respectively) on a keyboard^15^. There were two kinds of conditions; one was a congruent condition where the color names correspond to the font colors and the other was an incongruent condition where the color names and font colors do not match. The difference in reaction times (RTs) between incongruent and congruent conditions was measured as the Stroop score. We used cut-offs at the RTs below the 200 ms and two standard deviations above the mean RTs^16^. Only the corrected responses were used to calculate Stroop scores.

**Attention network test.** This test is known to assess inhibition and selective attention^11^. There were four different types of cues such as a spatial cue, double cue, center cue, and no cue. In the spatial cue, a star was presented on the upper or lower part of a fixated cross positioned in the center of a computer monitor. In the double cue, two stars appeared on both upper and lower parts simultaneously. In the center cue, a star was shown in the center. The no cue showed nothing on the screen. After the cue disappeared, flanker arrows were presented on the upper or lower part of the cross fixation. Among the four types of cues, only the spatial cue was informative to participants since it indicated the position of the flanker arrows. There were three types of flankers; a neutral type, which contained only one arrow indicating a right or left direction; a congruent type, which had five arrows in a row pointing in the same direction (either right or left); and an incongruent type, which showed four arrows pointing in the same direction (e.g., right) and the middle arrow pointing the opposite direction (e.g., left). Using the keyboard, participants were required to press a right or left shift key corresponding to the direction of the middle arrow^17^. The attention network test score was calculated as follows (1)^18^. We used cut-offs at the RTs below 200 ms and two standard deviations above the mean RTs^16^. Only the corrected responses were used to calculate the attention network test score.

$Score of attention network test= \frac{{RT}_{Incongruent}-{RT}_{Congruent}}{{RT}_{Congruent}} (1)$

**Go/No-go test.** The Go/No-go test assesses an individual’s ability of inhibitory control^19^. The test started with a $2\times2$ array of four star-shaped blue objects. In P-Go condition participants had to press the right shift key on a keyboard only when the letter P was presented in one of the four positions in the array. On the other hand, in R-No-go condition, participants were instructed not to respond when the letter R was presented. This rule changed in another session, for which R-Go and P-No-go conditions were presented. The accuracy of the No-go conditions represented the Go/No-go score^19,20^.

**Supplementary References**

1 Benton, A. L. Differential behavioral effects in frontal lobe disease. *Neuropsychologia* **6**, 53-60 (1968).

2 Schwartz, S., Baldo, J., Graves, R. E. & Brugger, P. Pervasive influence of semantics in letter and category fluency: A multidimensional approach. *Brain and language* **87**, 400-411 (2003).

3 Baldo, J. V., Schwartz, S., Wilkins, D. & Dronkers, N. F. Role of frontal versus temporal cortex in verbal fluency as revealed by voxel-based lesion symptom mapping. *Journal of the International Neuropsychological Society* **12**, 896-900 (2006).

4 Kang, Y. W., Chin, J. H., Na, D. L., Lee, J. H. & Park, J. S. A normative study of the Korean version of Controlled Oral Word Association Test ( COWAT ) in the elderly. *Korean Journal of Clinical Psychology* **19**, 385-392 (2000).

5 Ruff, R., Light, R., Parker, S. & Levin, H. Benton controlled oral word association test: Reliability and updated norms. *Archives of Clinical Neuropsychology* **11**, 329-338 (1996).

6 Engle, R. W., Tuholski, S. W., Laughlin, J. E. & Conway, A. R. Working memory, short-term memory, and general fluid intelligence: a latent-variable approach. *Journal of experimental psychology: General* **128**, 309 (1999).

7 Kessels, R. P., van Zandvoort, M. J., Postma, A., Kappelle, L. J. & de Haan, E. H. The Corsi Block-Tapping Task: standardization and normative data. *Appl Neuropsychol* **7**, 252-258, doi:10.1207/S15324826AN0704_8 (2000).

8 Grant, D. A. & Berg, E. A behavioral analysis of degree of reinforcement and ease of shifting to new responses in a Weigl-type card-sorting problem. *Journal of experimental psychology* **38**, 404 (1948).

9 Puente, A. Wisconsin card sorting test. *Test critiques* **4**, 677-682 (1985).

10 Miyake, A. *et al.* The unity and diversity of executive functions and their contributions to complex "Frontal Lobe" tasks: a latent variable analysis. *Cogn Psychol* **41**, 49-100, doi:10.1006/cogp.1999.0734 (2000).

11 Diamond, A. Executive functions. *Annu Rev Psychol* **64**, 135-168, doi:10.1146/annurev-psych-113011-143750 (2013).

12 Lysaker, P., Bell, M. & Beam-Goulet, J. Wisconsin Card Sorting Test and work performance in schizophrenia. *Psychiatry Research* **56**, 45-51 (1995).

13 Afsaneh, Z. *et al.* Assessment of Selective Attention with CSCWT (Computerized Stroop Color-Word Test) among Children and Adults. *Online Submission* (2012).

14 Spreen, O. & Strauss, E. in *InAdministration, norms, and commentary* (Oxford University Press, 1998).

15 Chen, E. Y., Wong, A. W., Chen, R. Y. & Au, J. W. Stroop interference and facilitation effects in first-episode schizophrenic patients. *Schizophrenia Research* **48**, 29-44 (2001).

16 Whelan, R. Effective analysis of reaction time data. *The Psychological Record* **58**, 475-482 (2008).

17 Fan, J., McCandliss, B. D., Sommer, T., Raz, A. & Posner, M. I. Testing the efficiency and independence of attentional networks. *J Cogn Neurosci* **14**, 340-347, doi:10.1162/089892902317361886 (2002).

18 Westlye, L. T., Grydeland, H., Walhovd, K. B. & Fjell, A. M. Associations between regional cortical thickness and attentional networks as measured by the attention network test. *Cereb Cortex* **21**, 345-356, doi:10.1093/cercor/bhq101 (2011).

19 Bezdjian, S., Baker, L. A., Lozano, D. I. & Raine, A. Assessing inattention and impulsivity in children during the Go/NoGo task. *Br J Dev Psychol* **27**, 365-383, doi:10.1348/026151008X314919 (2009).

20 Halperin, J. M. *et al.* Differential assessment of attention and impulsivity in children. *Journal of the American Academy of Child & Adolescent Psychiatry* **27**, 326-329 (1988).
